# Review: Livestock disease resilience: from individual to herd level

**DOI:** 10.1016/j.animal.2021.100286

**Published:** 2021-12

**Authors:** A. Doeschl-Wilson, P.W. Knap, T. Opriessnig, S.J. More

**Affiliations:** a The Roslin Institute, University of Edinburgh, Roslin Institute Building, Easter Bush EH25 9RG, Scotland, UK; b Genus-PIC, 24837 Schleswig, Germany; c Centre for Veterinary Epidemiology and Risk Analysis, School of Veterinary Medicine, University College Dublin, Veterinary Science Centre Belfield, Dublin D04 W6F6, Ireland

**Keywords:** Breeding, Disease resistance, Disease transmission, Infectious disease, Vaccination

## Abstract

Infectious diseases are a major threat to the sustainable production of high-producing animals. Control efforts, such as vaccination or breeding approaches often target improvements to individual resilience to infections, i.e., they strengthen an animal’s ability to cope with infection, rather than preventing infection *per se*. There is increasing evidence for the contribution of non-clinical carriers (animals that become infected and are infectious but do not develop clinical signs) to the overall health and production of livestock populations for a wide range of infectious diseases. Therefore, we strongly advocate a shift of focus from increasing the disease resilience of individual animals to herd disease resilience as the appropriate target for sustainable disease control in livestock. Herd disease resilience not only captures the direct effects of vaccination or host genetics on the health and production performance of individuals but also the indirect effects on the environmental pathogen load that herd members are exposed to. For diseases primarily caused by infectious pathogens shed by herd members, these indirect effects on herd resilience are mediated both by individual susceptibility to infection and by characteristics (magnitude of infectiousness, duration of infectious period) that influence pathogen shedding from infected individuals. We review what is currently known about how vaccination and selective breeding affect herd disease resilience and its underlying components, and outline the changes required for improvement. To this purpose, we also seek to clarify and harmonise the terminology used in the different animal science disciplines to facilitate future collaborative approaches to infectious disease control in livestock.

## Implications

Vaccination and breeding programmes that target improvement of herd disease resilience will lead to more effective control of many infectious diseases in production animals, as they not only reduce the impact of infectious pathogens on the health and production performance of individuals, but also on spread.

## Introduction

Infectious diseases are one of the most important threats to sustainable livestock production, especially for high-performance animals that are commonly reared in confined environments that foster pathogen transmission (Lindström et al., 2012, Craft, 2015). Climate change, antimicrobial resistance and recent modifications in agricultural practices and demography have been shown to exacerbate pathogen burden (Tomley and Shirley, 2009). Hence, more than ever, effective disease control is paramount for healthy farming systems, for national and international food security and for alleviating poverty in developing countries (Tomley and Shirley, 2009).

In addition to biosecurity, vaccination and selective breeding for increased disease resistance constitute the main preventive measures against infectious diseases in farmed livestock. However, neither current vaccines nor the animals’ natural genetic makeup usually confer full resistance to infection (Meeusen et al., 2007). Many veterinary vaccines, as well as breeding programmes, mitigate the impact of infection on the health and production performance of animals by improving their ability to cope with infection rather than preventing infection *per se* (Meeusen et al., 2007, Vale et al., 2016). In other words, they aim to increase the disease resilience of animals, which has been broadly defined as the ability of animals to cope with infectious challenge (Bisset and Morris, 1996) or, for production animals more explicitly as the ability of animals to maintain high production performance when challenged by infection (Albers et al., 1987). In a rapidly changing and increasingly connected world that fosters the circulation of existing and newly emerging pathogens across regions, countries and continents, a strong capacity to cope with infections is vital.

Epidemiological studies however draw increasing attention to a specific class of animals that appear to cope extremely well with infections, the so-called non-clinical carriers of infection (Table 1). These individuals are infected, and presumably infectious, for some period of time but without any clinical signs suggestive of disease. In other words, these individuals are both susceptible to infection and disease resilient. Whilst clearly beneficial for their own health and well-being, non-clinical carriers can impose a substantial threat to the health of other animals with whom they come into contact. The presence of non-clinical individuals has raised major challenges for the effective control of various infectious diseases in different species (e.g. Pastoret et al., 1992, Innocent et al., 1997, Alexandersen et al., 2002, Sánchez-Vizcaíno et al., 2012). For most diseases, accurate literature estimates for the proportion of non-clinical carriers of infections in livestock populations are rare. During the current COVID-19 pandemic, it has been reported that approximately 40–45% of infected people are asymptomatic (Oran and Topol, 2020). It is logical to also expect a high proportion of non-clinical carriers with some livestock infections.

**Table 1:** Glossary of terminology.

Term	Definition	Comments
Epidemiological concepts		
Infection	Colonisation of an animal’s body tissues and fluids by infectious pathogens and subsequent multiplication often leading to host innate, humoral and cellular immune responses (which potentially could result in damage)	
Disease caused by infectious agents	A disorder of structure or function of an infected animal	
Non-clinical carrier	An animal that is infected and infectious, but does not present with clinical signs	
Resistance to infection	Propensity to not become infected when exposed to infectious pathogens	Conversely, susceptibility to infection is the propensity to become infected when exposed to infectious pathogens
Resistance to disease	Propensity to not develop disease when infected	Conversely, susceptibility to disease is the propensity to develop disease when infected
Vaccine efficacy	The ability of a vaccine to provide protection against disease under ideal conditions (e.g. during a clinical trial).	An individual-level measure of vaccine effect, defined as the reduction in incidence of the target infection/disease in vaccinated participants compared to controls^1^
Vaccination effectiveness	A measure of the extent to which vaccination, when employed under field conditions, does what it is intended to do for a specified population^2^	A population measure of vaccine effect, capturing factors affecting both vaccine efficacy under field conditions and vaccine coverage (% of the population vaccinated). Often considered to include indirect vaccine effects on transmission^3^
Reproduction number *R* (*R* value)	Average number of secondary cases produced by a typical infectious individual during its infectious lifetime	If R < 1, the infection will decline and eventually die out.
Super-spreader	An infected individual that transmits an infection to an unexpectedly large number of other individuals	Super-spreaders are known to play an important role in the epidemiology of many infectious diseases

Animal production and breeding concepts		
Recoverability	The degree and rate of return to the health and performance state prior to exposure	Considers both the duration of disease and the potential for residual effects on health or production performance following disease recovery
Resilience (generic)	Capacity to be minimally affected by disturbances or to rapidly return to the state pertained before exposure to a disturbance^4^	Often also referred to as ‘robustness’ which is more commonly used to describe the combination of a high production potential with high resilience^5^
Disease resilience (individual, herd or system)	(1) capacity to be minimally affected by exposure to infection or to rapidly return to the pre-exposure state (2) ability of animals to maintain high production performance when challenged by infection 6	Adapted from the generic definition above
Component traits of individual disease resilience		These are direct effects, acting on the resilience of the individual itself
Disease resistance	Ability of the individual to inhibit or limit within-host pathogen replication^7^	This definition encompasses the epidemiological concepts of resistance to infection. There is inconsistence in the use of this term in the animal breeding literature, where it has referred to resistance to infection, to resistance to disease, or to mortality following exposure
Disease tolerance	Ability of an infected host to reduce the impact of infection on performance and health, i.e. maintain high health or production performance at a given within-host pathogen load^5^	Tolerance can only be expressed once an animal has become infected and therefore expression of tolerance is conditional on susceptibility to infection. Tolerance encompasses the epidemiological concept of resistance to disease, as well as recoverability

Component traits of herd disease resilience		
Direct effects, affecting an individual’s own fitness, health and performance		
Individual disease resilience	As defined above	The component traits include both resistance and tolerance (as defined above)
Indirect effects, i.e., additional components relating to environmental pathogen load, with the potential to affect the health and performance of susceptible herd members		
Susceptibility to infection	As defined above, under epidemiological concepts	This is both a direct effect (as it affects the individual’s own health and performance) and an indirect effect (as only susceptible individuals can become infected and transmit infections, thus affecting the health and performance of herd members)
Magnitude of infectiousness	Propensity of an infected individual to transmit infection to a typical (average) susceptible individual	Also referred to as host ‘infectivity’^8^; typically relates to the nature and amount of pathogen shedding per unit of time
Duration of infectious period	The time period over which an animal is infectious	Typically related to the duration of pathogen shedding. It is equal to the inverse of the recovery rate if the duration of the infectious period and the duration of disease are equal

1 Dean et al. (2019).

2 Porta (2014).

3 Shim and Galvani (2012).

4 Colditz and Hine (2016).

5 Knap and Doeschl-Wilson (2020).

6 Albers et al. (1987).

7 Råberg et al., 2009, Bishop, 2012.

8 Velthuis et al., 2002, Lipschutz-Powell et al., 2012.

For farmed animals, the role of these disease-resilient individuals in the spread and persistence of infections in a population provokes a potential re-thinking of our current approaches to improve livestock disease resilience. Important questions to be posed include: “How do disease-resilient individuals affect the environmental pathogen landscape?” and “Is there a risk that improvements in disease resilience, as opposed to resistance to infection, might inadvertently increase pathogen spread and hence jeopardises disease resilience at the population level, i.e., ‘herd disease resilience’?”. It is timely to consider the potential impact of current approaches to improve disease resilience of individual animals, both with respect to the environmental pathogen load and to population-level disease severity.

In this paper, we approach the above questions by advocating a more explicit shift from improved disease resilience of individual animals to herd disease resilience as the appropriate target for sustainable disease control of livestock. We demonstrate that typically herd disease resilience is not just the average resilience of its herd members. In particular, for infections transmitted primarily through infectious pathogens shed by infected herd members, herd resilience also contains additional components including the environmental pathogen load, individual susceptibility to infection and the magnitude and duration of infectiousness among those individuals that do become infected. We introduce various underlying animal intrinsic component traits of herd disease resilience and review what is known about how vaccination or selective breeding affects herd resilience and its underlying components. We propose future avenues towards more effective vaccination and breeding programmes for improving herd resilience.

This review also aims to unite concepts and research findings from diverse research disciplines (e.g. animal production science, infection and immunity, epidemiology, vaccinology, animal breeding), which often differ in the terminology used. We therefore provide a glossary of terms used in this perspectives paper (Table 1).

## Herd resilience instead of individual resilience as a target for effective disease control

Resilience can be an attribute of an individual animal (*individual resilience*), a herd (*herd resilience*) or an entire production system (*system resilience*) (Table 1). In the context of livestock production, disease resilience has been broadly defined as the ability of an animal, a herd (a group of animals) or an entire production system (several herds) to maintain high production performance in the face of pathogen challenge (after Bisset and Morris, 1996). More specifically, resilience is the capacity to be minimally affected by exposure to infectious pathogens or to rapidly return to the pre-exposure state (after Colditz and Hine, 2016).

Quantitatively, the disease resilience of a production animal can be measured in terms of the reduction in health, fitness or production performance (reduced from the level expressed in the absence of infection) when exposed to infectious pathogens (Bisset and Morris, 1996). In other words, more resilient individuals deviate less from their health, fitness or performance potential. The magnitude of reduction depends on factors relating to the host (e.g. the individual's resistance and tolerance, as defined in Table 1), and on factors relating to the pathogen (e.g. the pathogen’s virulence and load that the animal is exposed to) (Knap and Doeschl-Wilson, 2020). Therefore, individual disease resilience is conventionally modelled as the reaction norm of the individual’s health or performance on environmental pathogen load (Knap and Doeschl-Wilson, 2020).

Individuals are known to vary genetically in their response to pathogens, and thus in their disease resilience. Incomplete vaccine coverage or heterogeneous vaccine response may further exacerbate this variation between individual herd members (Raadsma et al., 1996, Raadsma et al., 1999, Sanglard et al., 2020).

It is important to note that herd resilience does not usually equal the average individual resilience of the animals in the herd. Blanc et al. (2013) made an explicit distinction between herd resilience and individual resilience: “herd resilience depends on the adaptive capacity of the animals in the herd (i.e., on individual resilience), together with the management decisions that affect the performance trajectories and local environment of the animals“. In the context of diseases that are primarily caused by infectious pathogens shed by individuals sharing the same environment, the crucial component of this ”local environment of the animals“ is the pathogen load that individuals are exposed to (hereafter denoted as *environmental pathogen load*). Each infected animal can contribute to the environmental pathogen load, with the potential to indirectly affect the health and performance of susceptible, in-contact animals. Hence, herd resilience as a whole is more than the sum of its component parts (the resilience of all individuals in the herd) as it also contains a component that determines the transmission of infectious pathogens among animals within the herd. Importantly, when there is variation in individual disease resilience, strategies that only focus on increasing individual resilience (e.g., by vaccination or genetic selection) may not necessarily improve the resilience of the herd if they simultaneously increase the environmental pathogen load, as outlined further below.

**Fig. 1:**
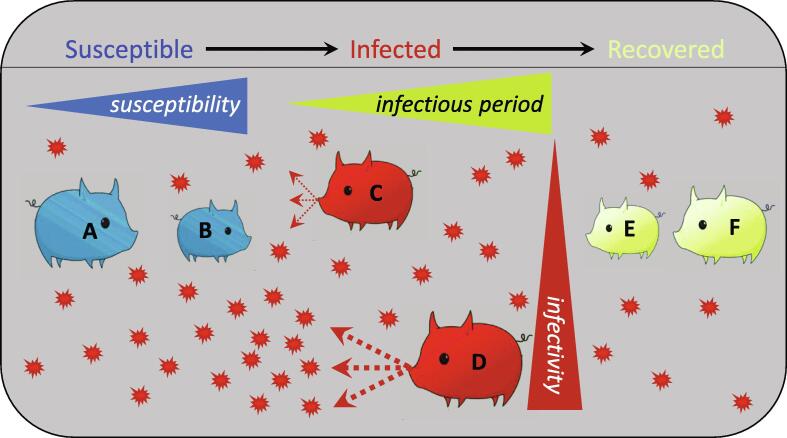
**A conceptual model of herd disease resilience for diseases primarily caused by infectious pathogens shed by infected herd members.** Herd disease resilience depends on (i) individual disease resilience (represented here by size: larger animals are more resilient, with the contributing mechanism outlined for each individual below) and on (ii) contribution of infected animals to the environmental pathogen load that susceptible animals in the herd are exposed to (represented here by the number of pathogen particles). Item (ii) is influenced by factors relating to the pathogen (innate characteristics including transmission potential) and by the host (susceptibility to infection, magnitude of infectiousness, duration of infectious period). In the context of the epidemiological SIR (Susceptible-Infectious-Recovered) model, where infected animals are assumed infectious until they reach the recovered state and have long-lasting immunity, these give rise to three epidemiological animal traits relating to pathogen transmission that influence herd resilience: *susceptibility* (among susceptible individuals: the probability of infection given exposure), *infectivity* (or magnitude of infectiousness; among infected individuals: the nature and amount of pathogen shedding per unit time) and the duration of the *infectious period*, with levels indicated in the figure by the animal's position relative to the corresponding wedges. Susceptible animal A has higher individual resilience than animal B because it is less susceptible to infection than animal B. As animal A is less likely to become infected given exposure, it is also less likely to contribute to the environmental pathogen load: its expected positive impact on herd resilience is greater than that of individual B. Infected animal D has a higher individual resilience than infected animal C as it is able to maintain high production performance (i.e., high tolerance) despite a long infected/infectious period. But animal D contributes more strongly to the environmental pathogen load due to both a longer infectious period and higher infectivity, therefore, it has a greater negative impact on herd resilience than animal C. Recovered animal E has lower individual resilience than animal F as it does not fully return to its pre-exposure state. Recovered animals no longer contribute to the environmental pathogen load, hence have no epidemiological impact on herd resilience. This figure only illustrates the herd resilience components for diseases that can be represented by an epidemiological SIR model. However, these concepts also apply to a wider range of epidemiological models, including, for example, SEIR (Susceptible-Exposed-Infectious-Recovered) or SIRS (Susceptible-Infectious-Recovered-Susceptible) models where individuals enter an exposed state before they become infectious or may become susceptible again after they have recovered.

Fig. 1 illustrates how individual resilience factors may interact with epidemiological components relating to environmental pathogen load, to determine herd resilience for a specific but relatively large range of infectious diseases that can be broadly represented by compartmental Susceptible-Infectious-Recovered (**SIR**) models. There, animals in a herd exposed to infectious pathogens may transition between three infection states: Susceptible (**S**), Infected and Infectious (**I**), and Recovered (**R**). The health, fitness or production performance of infected and recovered animals may be reduced relative to their performance in the absence of infection, and this depends partly on their multi-faceted individual resilience as mentioned above. The epidemiological component of herd resilience describes how individuals contribute to the environmental pathogen load, with potential implications for susceptible herd members. In the context of the SIR model (Fig. 1), each individual's relative contribution to the environmental pathogen load depends on (i) its *susceptibility* to infection (which also directly affects individual resilience), as well as (ii) its *infectivity* (i.e., magnitude of infectiousness, Table 1), and (iii) the duration of its *infectious period* (i.e., the inverse of its *recovery rate* in SIR models if the duration of the infectious period and the duration of disease are equal).

Animals are known to vary not only in their individual disease resilience but also in those epidemiological traits relating to environmental pathogen load (VanDerWaal and Ezwnea, 2016, Anacleto et al., 2019). Effective disease control that targets herd resilience could thus target improvement in individual resilience or any combination of the three above-mentioned epidemiological animal traits, crucially without negatively impacting the others. For example, it is not particularly useful to produce highly resilient animals that can cope with a high pathogen load if such animals also act as highly infectious super-spreaders that shed large amounts of pathogens (Gopinath et al., 2014, Beldomenico, 2020). Such individuals would have detrimental effects on the health and performance of less resilient animals in the herd. In contrast, approaches that reduce the environmental pathogen load (general biosecurity measures, vaccination, and animal breeding to reduce individual susceptibility to infection, infectivity and/or duration of the infectious period) may lead to increased herd disease resilience, even when the average disease resilience of individuals is low.

## Quantifying herd resilience traits and the impact of interventions on them

The above-mentioned herd resilience component traits are difficult to measure directly and thus may need to be estimated from available data. Knap and Doeschl-Wilson (2020) provide a review of current methods for estimating individual disease resilience. Estimates for the host traits related to pathogen transmission could be obtained by assessing pathogen shedding from infectious individuals (e.g., quantity and duration of detectable levels of pathogen in blood, tissues or faeces or saliva (Pileri and Mateu, 2016, Tsairidou et al., 2018). However, such measures are not always available. Also, they may not necessarily correspond to actual transmission patterns, which also depend on individual contact behaviour and environmental characteristics (Anderson and May, 1991). Instead, appropriate experimental or field study designs, coupled with statistical inference methods, may provide more accurate estimates of individual susceptibility and infectiousness directly from measurements of individual infection or disease status (Longini et al., 1998, Aznar et al., 2011, Pooley et al., 2020).

The transmission of infections, and the impact of interventions, are often quantified at a population level. The measure used for this purpose is the reproduction ratio *R*, which is defined as the average number of secondary cases produced by a typical infectious individual during its infectious lifetime (Diekmann et al.,1990). All disease control strategies that aim to reduce transmission aim to reduce *R* to a threshold value below 1, when infection will decline and eventually die out. It is commonly known that individuals can strongly differ in their contribution to *R,* through variation in susceptibility and magnitude and duration of infectiousness (Lloyd-Smith et al., 2005, VanDerWaal and Ezwnea, 2016, Anacleto et al., 2019). Estimation of R, and of individual contribution to it and how these are affected by interventions, requires detailed knowledge about pathogen-specific transmission patterns and expertise in epidemiological modelling and statistical inference techniques (see e.g. Diekmann et al., 1990, Lloyd-Smith et al., 2005).

## Effects of vaccines on herd resilience

Vaccination is one of the key tools available to control infectious diseases in livestock. Based on recent estimates, vaccines are available for over 400 diseases affecting mammals, birds and fish (Knight-Jones et al., 2014). Within the livestock production sector, some of the most widely used vaccine applications include those against Foot-and-Mouth Disease (**FMD**) in cattle, sheep, goats and pigs, the Porcine Reproductive and Respiratory Syndrome (**PRRS**) in pigs and Marek’s disease in poultry (Knight-Jones and Rushton, 2013, Tizard, 2021, Witter, 2001). In general, vaccinations improve animal disease resilience by either reducing or eliminating the adverse effects that infection causes on health and survival or productive performance (Knight-Jones et al., 2014). Whilst most vaccines reduce the probability of disease when infected (Table 1), surprisingly few livestock vaccines have been shown to protect animals from either becoming infected or from transmitting pathogens to other animals.

### Vaccine efficacy and effectiveness in the context of herd resilience

The degree to which a vaccine boosts the disease resilience of individuals, the so-called *direct effect of vaccination*, is routinely assessed in vaccine efficacy studies, which are compulsory for licensing a vaccine. The efficacy of a veterinary vaccine is evaluated as “the ability of the vaccine to give protection against adverse effects of the infection to the vaccinated animal” (Pastoret, 1997). It is typically assessed through challenge studies in which cohorts of vaccinated and non-vaccinated individuals are infected (usually through individual inoculation) with the target pathogen under controlled experimental conditions. The health and performance status of these two cohorts is subsequently compared.

Vaccination effectiveness refers to the performance of the vaccine under real-world conditions (Shim and Galvani, 2012), and accounts for many factors that may compromise vaccine efficacy on farm (e.g., timing of vaccination, physiological state of the animals, variation in animals’ vaccine responsiveness, pathogen strain variation, etc.) and the vaccination coverage rate (the percentage of the population receiving and responding to the vaccine, frequency of vaccination in relation to population and infection dynamics). In real-world conditions, there is the potential to protect unvaccinated individuals through the presence of vaccinated individuals in a population, through so-called *indirect effects of vaccination* on herd members (Shim and Galvani, 2012). In contrast to direct effects, these indirect vaccine effects are mediated by intervention-induced changes in pathogen transmission (Halloran et al., 2010, Bailey et al., 2020). More explicitly, a vaccine is considered as effective if it can reduce within-host pathogen burden (e.g., through increasing host resistance) and pathogen shedding (e.g., through reducing host infectivity or the duration of the infectious period), and prevent or alleviate disease-induced clinical signs (e.g., through improving host tolerance), thus improving the general health conditions of the exposed animals (Meeusen et al., 2007). Vaccine effectiveness thus targets herd resilience as it considers the direct vaccine effects on individuals’ own resilience as well as the indirect effect of vaccinated individuals on the performance of others through their contribution to the environmental pathogen load that others are exposed to (Fig. 1).

To date, there has been limited consideration of the indirect effects of vaccination for animal health and production. Indeed, there is only a limited literature that explicitly considers the effect of vaccines on pathogen transmission. This is in large part because an assessment of indirect vaccine effects is not required for vaccine authorization, and because different methodologies are required to estimate these effects (Longini et al., 1998, Orsel et al., 2007).

### Insights from case studies

There is some published information about the epidemiological effects of vaccination in animal populations which provides valuable insights into how vaccines may affect herd resilience. Some of these insights for different species are considered below.

#### Bovine tuberculosis in badgers and cattle

Bovine Tuberculosis (**bTB**, caused by infection with *Mycobacterium bovis*) is a zoonotic disease of bovidae (Banos et al., 2017). Badger to cattle transmission is one of the reasons contributing to difficulties faced in seeking to eradicate bTB from the United Kingdom and Ireland (Allen et al., 2018). The only licensed vaccine currently available and shown to confer protection against *M. bovis* is the live attenuated Bacille Calmette Guérin (**BCG**) (Hope and Villarreal-Ramos, 2008). Application of this vaccine to cattle is however forbidden under international and EU law because it is not possible to distinguish, in a diagnostic sense, between vaccinated and naturally infected cows. Instead, BCG vaccination of badgers is being used to control *M. bovis* infection in that wildlife species (Aznar et al., 2018), with evidence of beneficial downstream effects on bTB prevalence in cattle (Martin et al., 2020). A BCG badger vaccination field trial conducted in Ireland showed that susceptibility to natural exposure with *M. bovis* was reduced in vaccinated compared to placebo treated badgers, with a vaccine efficacy for susceptibility of 59% (Aznar et al., 2018). However, the trial revealed a complete lack of vaccine effect on the infectivity of vaccinated infected badgers, implying that vaccinated badgers were equally infectious as non-vaccinated badgers when naturally infected with *M. bovis*. This lack of indirect vaccination effects on *M. bovis* transmission raises demands on vaccination coverage in badger populations to achieve the desired vaccine effectiveness for eradicating bTB in badgers and cattle (Aznar et al., 2018).

#### Foot-and-mouth disease in ruminants and monogastrics and Porcine Reproductive and Respiratory Syndrome in monogastrics

Foot-and-Mouth Disease, affecting cloven-hooved animals including cattle, sheep, goats and pigs, and PRRS in pigs, constitute two economically important livestock diseases for which the pathological and epidemiological effects of vaccines have been assessed in several experimental and field studies. Both diseases are endemic in many countries worldwide, and although mortality is relatively low, morbidity and infection-induced production losses are high (Knight-Jones and Rushton, 2013, Nathues et al., 2017). Further, the presence of these diseases has important trade implications. Disease control in many areas where FMD or PRRS is endemic is generally implemented by means of regular mass vaccination, which has achieved noticeable success in reducing disease-associated production losses (Knight-Jones et al., 2014, Linhares et al., 2015). However, vaccination programmes have also shown variable and often limited success in reducing infection prevalence and have raised concerns about the epidemiological consequences of vaccination and urgent demand for more effective vaccines (Parida, 2009, Nan et al., 2017). This is particularly the case for the current modified live vaccines, which have caused several safety concerns with regard to the shedding of the modified live virus, potential recombination with circulating field strains or reversion to virulence.

Existing PRRS or FMD vaccines rarely confer sterilizing immunity for heterologous challenge strains, and thus likely only offer limited protection from infection for the cocktail of circulating virus strains that pigs are typically exposed to in the field (Parida, 2009, Nan et al., 2017). Using a comprehensive modelling framework to assess vaccine effectiveness applied to PRRS, Bitsouni et al. (2019) demonstrated that vaccines that offer no or limited protection from infection can still substantially reduce pathogen transmission and even achieve a value for the reproduction number, *R*, below one if vaccine coverage is high and the vaccine sufficiently reduces host infectivity or speeds up recovery. Numerous FMD and PRRS vaccine trials have shown that vaccinated animals challenged with a heterologous virus strain usually have a several fold reduction in shedding of that strain in comparison to unvaccinated control animals, and that virus loads in blood and other tissues more quickly fall to undetectable levels in vaccinated animals (Parida, 2009, Pileri and Mateu, 2016). Transmission experiments generally confirm that these vaccine effects translate into reduced FMD or PRRS virus transmission, and thus increased herd resilience under natural challenge conditions (see e.g., Orsel et al., 2007, Eblé et al., 2008, Van Roermund et al., 2010 for FMD and Pileri and Mateu, 2016, Chase-Topping et al., 2020 for PRRS). However, there is considerable variation between these experiments with regard to the nature and actual amount of reduction in infectious disease transmission, with *R* values often still remaining considerably greater than one in vaccinated groups. For example, Chase-Topping et al. (2020) reported an estimated *R* value well above one in vaccinated pigs with the vaccine primarily reducing the duration of virus shedding of infected pigs. In contrast, in a study by Rose et al. (2015) *R* was reduced to a value below one by the vaccine, simultaneously reducing the duration of shedding as well as the estimated transmission rate. It would be of benefit if future vaccine evaluations were to disentangle the vaccine effects relevant to individual and herd resilience traits, as also indicated in the modelling framework published by Bitsouni et al. (2019). In particular, closer investigation is needed of the role of infected individuals without clinical signs (i.e., non-clinical carriers) on the transmission of infection, noting that the prevalence of non- or subclinical individuals may increase as a consequence of vaccination (Horter et al., 2002, Paton et al., 2018).

#### Marek’s disease in poultry

There is increasing awareness that vaccination may not only affect the host but may also alter the pathogen landscape. Evolutionary theory predicts that leaky vaccines, i.e., those that allow the host to survive but do not prevent pathogen spread, may drive pathogen evolution towards increased virulence, thus jeopardising individual resilience in the long-term (Gandon et al., 2001). This is particularly pertinent for Marek’s disease (**MD**) in chicken, where step jumps in virulence of field strains of MD virus were repeatedly observed shortly after release of new vaccines (Witter, 1997). As MD vaccines prevent disease and death but allow viral replication and transmission, Read et al. (2015) have argued that such leaky vaccines alter the balance of selection between pathogen transmission and virulence by allowing more virulent strains to be transmitted at reduced cost. Whilst plausible, direct empirical evidence from transmission studies that vaccines drive pathogen evolution to higher virulence is still lacking. Recent transmission experiments revealed that pathogen transmission from vaccinated hosts can cause dose-dependent reduction in pathogen virulence (Bailey et al., 2020). The study showed that non-vaccinated infected contact birds were less likely to develop disease and shed significantly less virus when exposed to vaccinated rather than non-vaccinated infected shedder birds. These results emphasise the importance of also considering the indirect effects of vaccination on non-vaccinated herd members, highlighting the urgent need for more elaborate transmission experiments and field studies to fully determine vaccine effects on MD transmission, virus evolution and herd resilience in the long-term.

#### Infectious diseases in aquaculture

Vaccine use is also becoming more widespread to prevent disease in aquaculture (Dadar et al., 2017), and has been a key reason for the success of salmon cultivation (Sommerset et al., 2005). In aquaculture, vaccine efficacy is mostly assessed through reduction in mortality rates in vaccinated populations (Dadar et al., 2017). These often involve so-called bath-challenge experiments, where vaccinated or naïve fish are exposed to antigens released in water, which likely mimic natural infection and transmission more closely than would occur with inoculation methods as used during efficacy studies in other species.

Very little is known about the impact of vaccines on pathogen transmission in aquaculture, as *in vivo* diagnostics of the infection status rarely exist for these species, and transmission experiments are extremely rare. Recent reviews report that fish vaccines often suffer from low efficacy, i.e., they lead to noticeable reduction but not to zero mortality, indicating that many fish vaccines are leaky and do not prevent pathogen transmission (Adams, 2019, Bøgwald and Dalmo, 2019). This was confirmed in a recent transmission trial that assessed vaccination effects on the transmission of Infectious Salmon Anaemia (**ISA**) virus in Atlantic salmon, which showed that ISA vaccination reduced, but did not prevent, pathogen transmission and mortality (Chase-Topping et al., 2021). The study also demonstrates that even small-scale transmission experiments can quantify vaccine effects on the transmission of infection and herd resilience parameters from mortality records alone when coupled with epidemiological models, as predicted by simulation studies (Pooley et al., 2020).

In summary, recent studies have demonstrated an indirect vaccine effect in some vaccines that were primarily designed to increase individuals’ resilience to infectious disease. Some vaccines may add additional benefits, relevant to herd resilience, by reducing the transmission of infectious pathogens both through increased resistance to infection and reduced infectiousness. For most vaccines however, the epidemiological benefits and evolutionary risks are poorly understood. Routine implementation of transmission experiments, coupled with statistical and epidemiological models in vaccine evaluation studies, would be extremely useful to determine optimal vaccines that simultaneously boost individual and herd resilience to infectious diseases. Furthermore, recent developments in pathogen genome sequencing and phylogenetics should provide valuable insights into the effects of vaccination on the pathogen landscape and the corresponding impact on herd resilience in the long-term (Chong et al., 2010, Hadfield et al., 2018).

## Effects of animal breeding on herd resilience

There is overwhelming empirical evidence that animals differ genetically in their response to infectious pathogens (Bishop, 2010). However, for the majority of prevalent diseases in production animals caused by infectious pathogens, and similar to vaccination, the natural genetic make-up rarely offers full protection to animals from becoming infected. Thus, animals with high genetic resistance can be susceptible to infection when exposed, can transmit the pathogen, and contribute to the environmental pathogen load. Hence the epidemiological components introduced in Fig. 1 also likely play an important role for genetic improvement of herd disease resilience. However, this has not yet been fully capitalised by the livestock breeding community.

### A brief overview of current breeding practices to control infectious diseases

Genetic disease control, through selective breeding of animals that genetically respond more robustly to infection, has long been proposed as a viable alternative or complement to vaccination (Bisset and Morris, 1996, Stear et al., 2001). Whereas vaccine trials aim to minimise heterogeneity between animals (Velthuis et al., 2003), animal breeding relies on and utilises genetic variation to improve the host response to infection from one generation to the next. Despite considerable research investment, to date the livestock breeding sector applies little explicit selection for disease resilience (see Knap and Doeschl-Wilson, 2020 for a review and future outlook on this). The primary focus has been on improving individual disease resistance (see Table 1 under animal production and breeding concepts), although the exact definition of ‘disease resistance’ differs in the animal breeding literature across host and pathogen species, with different implications for herd resilience, as will be outlined below.

Regardless of the exact definition used, breeding for disease resistance has been implemented in most livestock and aquaculture species (Bishop and Woolliams, 2014, Houston, 2017), albeit only for a small number of infectious diseases relative to those tackled with vaccination. Successful examples include the national sheep breeding programmes for scrapie resistance in several European countries, or marker-assisted selection for infectious pancreatic necrosis (IPN) resistance in Atlantic salmon and for *E. coli* resistance in pigs. These programmes each resulted in a reduced infection prevalence to acceptably low levels within less than 10 years of selective breeding (Boelaert et al., 2016, Hjeltnes et al., 2018, Luther, 2018). However, progress with each of these diseases can be attributed to the fortuitous but rare discovery of a single gene with a large effect on disease resistance. For the more common situation of polygenically controlled disease resistance (where resistance is primarily controlled by many host genes with small effects; see for example diseases listed in Table 2), genetic improvement generally happens at a slower rate, with less obvious effects on disease prevalence (and thus on herd resilience) in subsequent generations. However, as shown in experimental studies for gastro-intestinal parasite infections in sheep, genetic selection for the polygenically controlled faecal egg counts (**FEC**) can have a larger and more persistent effect on reducing FEC in the host, as well as on worm contamination on pasture, than vaccination or other non-genetic control measures (Eady et al., 2003). Also, genetic selection for traits that are controlled by many host genes, such as FEC in the example above, is likely to impose lower risk for pathogen evolution and thus long-term herd resilience than, for example., vaccines or genetic selection methods that target few genes (Woolaston et al., 1992, Kemper et al., 2009).

**Table 2:** Examples for genetic selection for disease resistance considered in current breeding programmes, and their potential effect on pathogen transmission and herd resilience.

Disease & Species	Resistance phenotype	Characteristics of a resistant animal	Effects on pathogen transmission and herd resilience
Bovine tuberculosis (bTB) in cattle	Binary infection status from in vivo diagnostic skin test applied in herds exposed to bTB	Less likely to have a positive test result when exposed to bTB^1^	Some uncertainty in whether genetically more resistant cows are less likely to become infected and to transmit bTB^2^
Marek’s disease (MD) in poultry	Clinical signs (e.g. lameness, lesions) and mortality after exposure to MD virus in challenge trials	Less likely to develop clinical MD and subsequently die^3^	Chicken with high genetic resistance can still become infected and transmit the MD virus^1^. Unknown if breeding for MD resistance reduces MD virus transmission
Viral and bacterial infections in Atlantic salmon	Binary survival or time of death after pathogen exposure in challenge trials	Less likely to die when exposed to the pathogen in consideration^4^	Unknown whether fish considered genetically more resistant have greater disease resistance or tolerance, or both. Unknown if breeding for disease resistance reduces pathogen transmission.
Gastro-intestinal parasite resistance in ruminants	Parasite egg count in faeces (Faecal egg counts, FEC)	Lower FEC may reflect the ability of an animal to limit parasite establishment, growth, fecundity and/or shedding^5^	Breeding for disease resistance reduces parasite shedding and thus the environmental parasite load, with beneficial effects on herd resilience^6^
Porcine Reproductive & Respiratory Syndrome (PRRS) in pigs^7^	Blood viral load of pigs over 21 day infection period after inoculation with the PRRS virus	Pigs that carry the beneficial (GBP5) allele associated with greater natural^8^ PRRS resistance are still susceptible to PRRS virus infection by inoculation, but tend to have lower virus replication^9^	Currently unknown if pigs that carry the GBP5 resistance allele are more resistant to infection and less infectious when infected in natural challenge conditions. Hence the effect of genetic selection on PRRS virus transmission is unknown.

1 Banos et al. (2017).

2 Bishop and Woolliams (2014).

3 Bacon et al. (2001).

4 Ødegård et al. (2011).

5 Bisset and Morris (1996).

6 Stear et al. (2001) and Eady et al. (2003).

7 Currently included in genetic evaluations, but not explicitly included in the formal selection criteria.

8 As opposed to resistance through gene editing, which confers full resistance to PRRS virus infection.

9 Boddicker et al. (2014).

The long-term effects of breeding for increased resistance (and for any other trait) are cumulative and permanent. But because the benefits only occur in subsequent generations, breeding is mostly considered as a complementary control strategy to vaccination or other short-term control measures (Bishop and Woolliams, 2014).

### Impact of selective breeding for disease resistance on herd resilience

**Fig. 2:**
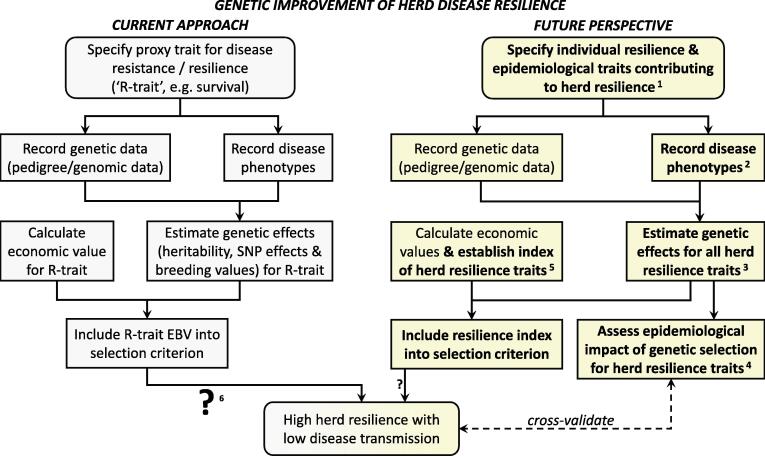
**Scheme of a potential future breeding programme with improved herd disease resilience as its breeding objective, contrasted to current approaches focusing on improved (individual) disease resistance or resilience.** Current approaches are in grey (left), proposed future approach is in yellow (right). Innovative components are highlighted in bold. Abbreviations: SNP = Single Nucleotide Polymorphism; EBV = estimated breeding value.**^1^**These herd resilience traits may include individual resilience as well as host traits controlling pathogen transmission, such as susceptibility to infection, infectivity and duration of the infectious period (see Table 1 and Fig. 1). **^2^**The disease phenotypes collected may be the same as in the current breeding programmes, or new measures as new diagnostics become available. **^3^**Improved statistical models that incorporate genetic and epidemiological theory will be used to estimate genetic effects for the diverse herd resilience traits, and their genetic relationship, from observable disease phenotypes (see section ‘Estimating genetic effects for the epidemiological animal traits’ for further information).**^4^**This can be assessed using genetic-epidemiological simulation models (see section ‘Integrating epidemiological models into quantitative genetics models’ for further information). **^5^**It may not be necessary to explicitly include all herd resilience traits into the selection index depending on their heritabilities, genetic correlations and prediction accuracies. **^6^**The size of the question mark symbolises the degree of uncertainty in the outcome of the decision with respect to whether the breeding objective is achieved. We expect this uncertainty to reduce drastically for breeding programmes that include epidemiological components.

In contrast to the vaccine literature, there are very few published studies that have quantified the effects of selective breeding on pathogen transmission from real data (e.g. Eady et al., 2003, Chase-Topping et al., 2020). Hence, the exact realised impact of selective breeding for disease resistance on herd resilience is mostly not known (Fig. 2, left hand panel).

Uncertainty in these effects is exacerbated by the fact that in the animal breeding literature, the term “disease resistance” is not always consistently defined. It often also lacks equivalence to epidemiological concepts and can refer to different components of herd resilience (see Table 1). Given the large data volumes required to estimate genetic parameters with sufficient accuracy for breeding purposes, the specific breeding goal trait representing disease resistance is often determined by the resistance phenotype, i.e., the trait that can be measured. The diversity of resistance phenotypes used may lead to different outcomes for herd resilience. Table 2 shows some examples of resistance phenotypes considered in current breeding programmes and anticipated effects on pathogen transmission and herd resilience. These illustrate that the success of breeding programmes in reducing the transmission of infectious pathogens depends strongly on the resistance phenotype used, and to a large extent is unknown. Consequently, without explicit consideration of all herd resilience components, in particular those relating to pathogen transmission, selective breeding for individual disease resistance as currently practised, is likely sub-optimal for improving herd resilience.

### New approaches enabling selective breeding for improved herd resilience

Fig. 2 illustrates the changes to a breeding programme required to shift its breeding objective from improvement of individual disease resistance (which is often poorly defined) towards improvement of herd resilience for diseases primarily caused by infectious pathogens shed by infected herd members. This shift entails two fundamental methodological changes to current approaches: (i) the integration of epidemiological models into the quantitative genetics machinery, and (ii) replacement in the selection criterion of current individual disease resistance traits with an index of the herd resilience traits introduced above. The key benefit arising from these changes is that such a breeding programme can improve herd resilience based on predictions of selection response in the individual animal traits contributing to herd resilience as well as in disease prevalence at the herd level, which can be validated with real data.

The proposed changes in breeding programmes will involve new demands for data recording and computational methods for estimating genetic and epidemiological effects, as outlined below. Recent advances in estimating (and genetically improving) individual disease resilience through their resistance or tolerance have been described elsewhere (e.g. Knap and Doeschl-Wilson, 2020). Hence we focus here on the components of herd disease resilience relating to pathogen transmission.

#### Integrating epidemiological models into quantitative genetics models

Breeding programmes are underpinned by a strong body of quantitative genetics theory to estimate genetic and economic effects associated with the inclusion of specific traits in the selection criterion (Fig. 2). For example, once genetic parameters (e.g., heritability, prediction accuracy) for a particular proxy trait for disease resistance or resilience (denoted ‘R-trait’ in Fig. 2) have been estimated, the well-known ‘breeder’s equation’ can be used to obtain an estimate for the expected change in the average R-trait value over successive generations of selective breeding (Lush, 1937). However, quantitative genetics theory alone cannot predict how genetic selection for the R-trait affects pathogen transmission. This is because the relationship between the R-trait and pathogen transmission is non-linear and depends on the animals’ susceptibility to infection and the infectivity and duration of infectious period, when infected. Consequently, the impact on pathogen transmission and therefore on herd resilience of breeding programmes that build on quantitative genetics theory alone is difficult to predict (as denoted by the large question mark in the left hand panel of Fig. 2).

Bishop and Stear (1999) were the first to integrate epidemiological theory into quantitative genetics models. Using gastro-intestinal parasitism in sheep, they demonstrated that the breeder’s equation vastly underpredicts the response to selection for disease resistance in terms of the population’s parasite load when epidemiological effects are ignored. Various genetic-epidemiological models have been developed since to predict how selective breeding reduces the incidence or prevalence of infectious diseases over successive generations in various animal species, including fish (e.g., MacKenzie and Bishop, 2001, Nieuwhof et al., 2009, Gharbi et al., 2015, Raphaka et al., 2018). However, whilst these models have proven extremely useful for demonstrating and quantifying the potential epidemiological effects of selective breeding, they have not been incorporated into current breeding programmes. This is because these models require as input genetic parameter estimates for the underlying animal traits affecting pathogen transmission (e.g., susceptibility to infection, infectivity, duration of infectious period), which until recently have been difficult to estimate from existing data.

#### Estimating genetic effects for herd resilience traits related to pathogen transmission

Some of the disease resistance phenotypes used to date (see e.g. Table 2) may be considered as proxies for susceptibility to infection. In contrast, nematode FEC, the main resistance phenotypes used in genetic control of gastro-intestinal parasite infections in sheep (Eady et al., 2003), could be equally considered as a phenotype for infectivity as it reflects the amount of parasites shed by an infected sheep into the environment. However, for the majority of infectious diseases, genetic parameter estimates for infectivity or the duration of the infectious period, or for recovery rate as a proxy of the latter (on the assumption that the duration of the infectious period and the duration of disease are equal), are extremely scarce. In particular, despite substantial evidence that individuals vary in infectivity (e.g. Velthuis et al., 2002), and that highly infectious super-spreaders are a common phenomenon (Lloyd-Smith et al., 2005), empirical evidence that infectivity is partly under genetic control only exists for few infectious diseases (e.g., Albers et al., 1987, Anacleto et al., 2019). A main reason for this paucity of estimates, and their correlations, is that the herd resilience traits relating to pathogen transmission can rarely be measured directly (see section *Quantifying herd resilience traits and the impact of interventions on them)*. Hence genetic parameter estimates for these traits need to be inferred from data that *can* be measured, such as the resistance phenotypes used in current studies (see e.g., those listed in Table 2). Bishop and Woolliams (2014) outline how the genetic signal for these resistance phenotypes can be masked by various sources of noise inherent in these data. Theoretical and empirical studies indicate that disentangling genetic effects for susceptibility to infection from those relating to infectivity or recovery either requires additional data or theoretical expansions of the classical linear mixed model machinery. For example, in the case of gastro-intestinal parasite infections, measures related to parasite establishment or growth in the host, such as worm burden or worm length, in addition to the conventional FEC measures, may help to disentangle host genetic susceptibility from infectivity (Stear et al., 2001). Recent studies have expanded conventional quantitative genetics threshold models to enable joint genetic parameter estimation for susceptibility to, and recovery from, mastitis in cattle from routinely collected repeated measures of somatic cell counts (Franzén et al., 2012, Welderufael et al., 2016). These studies indicate that susceptibility and recovery are genetically different traits. Together, these studies indicate that disease data may comprise considerably more genetic variation that can be exploited for selective breeding than previously anticipated.

Estimating genetic effects for infectivity without direct individual measurements of pathogen shedding proves more difficult, because an individual’s infectivity affects the infection status of other individuals, and it is usually not known who infects whom (Lipschutz-Powell et al., 2012). Several alternative approaches have been proposed to estimate genetic susceptibility and infectivity effects (e.g. Anacleto et al., 2015, Biemans et al., 2017, Biemans et al., 2019), and more recently to estimate genetic effects and correlations for susceptibility to infection, infectivity and recovery rate simultaneously (Pooley et al., 2020). All of these incorporate quantitative genetics methodologies into epidemiological models or vice versa. Although further developments and validation of these methods is ongoing, results from both simulated and real data analyses suggest that it is possible to obtain reasonably accurate and unbiased estimates of genetic effects for these traits from repeated records of individuals’ infection and/or survival status given appropriate data structures (e.g., observations from multiple herds or groups of individuals, in the case of infectivity) (Anacleto et al., 2015, Biemans et al., 2017, Pooley et al., 2020). This paves the way for including epidemiological traits and prediction models into a data-driven breeding programme with improved herd disease resilience as its breeding objective, as shown in Fig. 2. Most livestock breeding programmes already practise multi-trait selection using the well-established selection index theory (Lin, 1978). Which of the above listed herd resilience traits should be included in the selection index depends largely on their heritabilities and genetic correlations with each other and with the other traits in the selection index. It also depends on how accurately the genetic merit for this trait, and for pathogen transmission can be predicted. The latter requires epidemiological prediction models.

### Emerging empirical insights of the epidemiological effects of selective breeding

Although still in their infancy, interesting new empirical insights for herd disease resilience have already started to emerge from combining genetic and epidemiological methods. For example, analysing endemic claw disease digital dermatitis (**DD**) data in dairy cattle, Biemans et al. (2019) demonstrated that it is possible to estimate genomic breeding values for the basic reproduction number (*R*_0_) for this complex disease with reasonable accuracy from a limited amount of field data, by accounting for genetic variation in susceptibility and infectivity. IPN virus transmission experiments in Atlantic salmon revealed that individuals carrying the beneficial allele for IPN resistance (i.e., conferring lower mortality following exposure) had both lower susceptibility to IPN virus infection and lower infectivity but also appeared to die sooner when infected (i.e., had shorter infectious periods) (Doeschl-Wilson et al., 2018). This extremely fortunate combination of allelic effects may partly explain the observed success of existing breeding programmes to drastically reduce IPN outbreaks within a few years of selection, exceeding expectations based on quantitative genetics theory (Hjeltnes et al., 2018). However, this fortunate combination does not always apply. In a similar transmission experiment for ISA virus in Atlantic salmon, animals that differed in their estimated genomic breeding value for ISA resistance (**GEBV**, based on mortality records from previous ISA virus challenge tests) did not differ significantly in their susceptibility to ISA virus infection (Chase-Topping et al., 2020). However, individuals with high resistance GEBVs tended to have lower infectivity and a higher chance to recover or survive the infection, indicating that selection for ISA resistance improves survival and reduces transmission of ISA virus infected fish. Interestingly, in this study the estimated genetic effects on virus transmission were stronger than the vaccine effects, indicating that vaccination alone may not always be the most efficient method to improve herd resilience.

## Some additional considerations

The concepts and methodologies for herd disease resilience outlined in this paper focus on infectious diseases for which individual animals make an important contribution to the environmental pathogen load, which then puts other herd members at risk of infection. Although a wide range of diseases affecting high-producing farmed animals are primarily caused by infectious pathogens shed by herd members, this is not always the case. Examples include situations where infectious pathogens are transmitted primarily by wild hosts (e.g., migratory birds for Avian Influenza or Newcastle disease, or red deer or feral cattle for tick-borne diseases in herds where animal to animal transmission is low, or insects in the case of Akabane virus infections in ruminants or other vector-borne diseases), or diseases caused by pathogens that lie dormant in the environment, often for extended periods (e.g., soil-borne diseases such as Anthrax in cattle and other herbivores). For these types of diseases, herd disease resilience would be more effectively improved by improving individual disease resilience and by protecting susceptible animals from infection through appropriate changes to the animals’ environment, where possible. Similar arguments may apply to diseases for which infectious pathogens are not the primary cause. For example, pink eye or eye cancer in cattle may be caused by commensal organisms that become pathogens due to a change in the physiological status of the host or due to environmental stressors (e.g., bright sunlight or high UV exposure) (Walker, 2007, Lakshmi et al., 2020). Other examples, for which the relative contributions of the epidemiological components to herd resilience would need to be re-evaluated include reproductive diseases where the exposure of susceptible individuals to infectious pathogens depends to a large extent on management decisions, such as e.g., those regarding the distribution of semen in artificial breeding programmes or the use of sires in syndicate mating schemes. In these cases, low infectivity of the chosen sires may be of even greater importance for herd resilience.

Perhaps the biggest challenge for increasing herd disease resilience constitute disease complexes that are not caused by a single aetiological agent, such as the bovine respiratory disease complex or fleece rot or foot rot in sheep. Transmission patterns may differ for the different pathogens involved in these complexes, and also the resilience, susceptibility and infectiousness of individuals may be pathogen type or strain specific. Consequently, the indirect effects of vaccination or selective breeding on pathogen transmission may be particularly difficult to quantify, and hence focusing on methods that increase individual resilience may be the most feasible approach in practice.

Similar considerations apply if the goal is to improve herd disease resilience for multiple types of pathogens at once (i.e., generic disease resilience), given that farm animals are usually exposed to multiple types of pathogens at any point in time. Indeed, many vaccination and genetic studies aim to identify and exploit immune response mechanisms that offer generic protection to range of pathogens (e.g., Gunia et al., 2018, Netea et al., 2020, Ballester et al., 2020). Approaches to estimate individual disease resilience to multiple pathogens have been proposed (e.g., Putz et al., 2019). More research into these heterologous genetic and vaccine effects on various pathogens and their interactions as well as on pathogen transmission is required to understand their true impact on herd resilience.

Improving herd disease resilience aims to simultaneously maintain high herd-level production performance whilst also reducing pathogen transmission. However, it may not always be the optimal or even most appropriate control strategy for infectious diseases. In particular, if the aim is to eliminate or eradicate a disease, more drastic interventions with a greater focus on reducing pathogen transmission, such as movement restrictions and culling of infected individuals and their potential contacts, may be required. Improvement of herd disease resilience can complement these and potentially prevent or reduce the need of such drastic interventions.

## Conclusions

Disease control efforts should focus on herd resilience rather than on individual resilience. For diseases primarily caused by infectious pathogens shed by herd members, the importance of improved evaluations of vaccination and breeding programmes with respect to their influence on pathogen transmission cannot be overemphasised. Evidence presented above shows that to date we have limited understanding of the impact of either vaccination or selective breeding on herd resilience, as the effects on pathogen transmission are not fully understood. In particular, the results from empirical and simulation studies suggest that the potential contribution of selective breeding to reducing the transmission of infectious pathogens and thus on herd resilience is not yet realised. Also, very few studies to date have assessed the combined effects of vaccination and selective breeding on infectious disease prevalence and impact, although both methods are frequently applied jointly. To remedy these shortcomings and design more effective vaccines or breeding goals for herd disease resilience, a paradigm shift of our current approaches to control of infectious diseases in livestock may be required. This shift would include routinely carried out transmission experiments or field studies coupled with (genetic-) epidemiological models to capture both the direct effects of selective breeding or vaccination on individual resilience to infectious diseases and the indirect effects on herd resilience mediated through pathogen transmission. In addition, assessment of vaccination or selective breeding for herd resilience traits on pathogen evolution is paramount for long-term success of these strategies. Progress in these areas requires closer collaboration between the disciplines of infection and immunity, animal breeding and epidemiology, including greater harmonisation in terminologies, as exemplified in this article.

## Ethics approval

Not applicable; this article only presents previously published experimental and field data.

## Data and model availability statement

No data or models were generated for this study; only results from already published data analyses and modelling studies are presented.

## Author ORCIDs

**Andrea Doeschl-Wilson:** https://orcid.org/0000-0002-2658-6973

**Pieter W. Knap:** https://orcid.org/0000-0001-7765-7842

**Tanja Opriessnig:** https://orcid.org/0000-0001-9642-0904

**Simon J. More:** https://orcid.org/0000-0002-4270-0385

## Author contributions

**ADW:** Conceived the study and composed the first draft.

**PK, TO** and **SM** contributed to all sections of the paper and read and approved the final version.

## Declaration of interest

The authors declare no conflict of interest.
